# Dietary Concentrate-to-Forage Ratio Affects Rumen Bacterial Community Composition and Metabolome of Yaks

**DOI:** 10.3389/fnut.2022.927206

**Published:** 2022-07-14

**Authors:** Simeng Yi, Dongwen Dai, Hao Wu, Shatuo Chai, Shujie Liu, Qingxiang Meng, Zhenming Zhou

**Affiliations:** ^1^State Key Laboratory of Animal Nutrition, College of Animal Science and Technology, China Agricultural University, Beijing, China; ^2^Qinghai Academy of Animal and Veterinary Sciences, Qinghai University, Xining, China

**Keywords:** yak, rumen, microbiota, metabolomics, concentrate-to-forage ratio

## Abstract

Changes in dietary composition affect the rumen microbiota in ruminants. However, information on the effects of dietary concentrate-to-forage ratio changes on yak rumen bacteria and metabolites is limited. This study characterized the effect of three different dietary concentrate-to-forage ratios (50:50, C50 group; 65:35, C65 group; 80:20, C80 group) on yak rumen fluid microbiota and metabolites using 16S rRNA gene sequencing and liquid chromatography-mass spectrometry (LC-MS) analyses. Rumen fermentation parameters and the abundance of rumen bacteria were affected by changes in the dietary concentrate-to-forage ratio, and there was a strong correlation between them. At the genus level, higher relative abundances of *norank_f__F082*, *NK4A214_group*, *Lachnospiraceae_NK3A20_group*, *Acetitomaculum*, and *norank_f__norank_o__Clostridia_UCG-014* were observed with a high dietary concentrate-to-forage ratio (*P* < 0.05). Combined metabolomic and enrichment analyses showed that changes in the dietary concentrate-to-forage ratio significantly affected rumen metabolites related to amino acid metabolism, protein digestion and absorption, carbohydrate metabolism, lipid metabolism, and purine metabolism. Compared with the C50 group, 3-methylindole, pantothenic acid, D-pantothenic acid, and 20-hydroxy-leukotriene E4 were downregulated in the C65 group, while spermine and ribose 1-phosphate were upregulated. Compared to the C50 group, Xanthurenic acid, tyramine, ascorbic acid, D-glucuronic acid, 6-keto-prostaglandin F1a, lipoxin B4, and deoxyadenosine monophosphate were upregulated in the C80 group, while 3-methylindole and 20-hydroxy-leukotriene E4 were downregulated. All metabolites (Xanthurenic acid, L-Valine, N-Acetyl-L-glutamate 5-semialdehyde, N-Acetyl-L-glutamic acid, Tyramine, 6-Keto-prostaglandin F1a, Lipoxin B4, Xanthosine, Thymine, Deoxyinosine, and Uric acid) were upregulated in the C80 group compared with the C65 group. Correlation analysis of microorganisms and metabolites provided new insights into the function of rumen bacteria, as well as a theoretical basis for formulating more scientifically appropriate feeding strategies for yak.

## Introduction

The yak (*Bos grunniens*), is one of the most important specieson the Qinghai-Tibet plateau and the basis of the survival of local herders (1). Grazing is the conventional means of yak feeding. However, this practice is degrading grassland on the Qinghai-Tibet Plateau as the number of yak herds increases (2). The grassland degradation coupled with the harsh climatic conditions of the Qinghai-Tibet Plateau imposes a stress on the growth of yaks (3). In recent years, with the improvement of the local economy, the yak feeding pattern has also changed. Large-scale farms have been established (4). The rationality of the diet is key to improving production performance of yaks. Improving nutritional management, especially the design of a reasonable concentrate-to-forage ratio diet, is crucial.

The rumen is one of the most important organs in ruminants. Rumen function has an important impact on the production of ruminants. Rumen fermentation benefits from abundant, diverse, and metabolically active bacteria. Rumen bacteria ferment feed and perform many complex metabolic activities. The bacteria also use the metabolites in the rumen for self-proliferation (5, 6). Changes in the dietary concentrate-to-forage ratio significantly affect the bacterial community in the rumen (7–9). Most research on the dietary concentrate-to-forage ratio focuses on the effect on the performance of ruminants. However, the mechanisms of the interactions of the rumen microbiota and metabolome with dietary concentrate-to-forage ratios are ambiguous. It is necessary to characterize the complex relationship between the rumen microbiota and metabolites in yak. The analysis of rumen microbiota and their metabolites using high-throughput sequencing *via* 16S rRNA sequencing technology and metabolomic techniques has received increasing attention. Liu et al. (10) confirmed that the rumen bacterial community of yak is dynamically affected by different types of feed and changes in the feed. The bacterial diversity of yak fed grain was lower than that of yak fed forage. This may be because there is much less crude fiber in grain than in forage. Similar results were reported by Xu et al. (11) in a study exploring the differences in rumen bacteria between grass-fed and grain-fed yaks. A comprehensive analysis of the composition of the yak rumen microbiota would provide important insights into microbially mediated metabolic processes.

Here, to test the hypothesis that different dietary concentrate-to-forage ratios affect the rumen microbiota and metabolites, a combination of 16S rRNA gene sequencing and liquid chromatography-mass spectrometry (LC-MS) analysis was employed to characterize the rumen microbiota and metabolites of yaks. Possible relationships between rumen microbiota and metabolites were explored to provide more fundamental knowledge about yak fattening.

## Materials and Methods

### Animals, Diets, and Feeding Management

The experiment was conducted from October to December at the breeding base in Zhaxi, Guinan County, Qinghai Province, China. Eighteen healthy male yaks, 3-years-old, in good condition, with similar body weight (164.9 ± 12.9 kg) were randomly divided into three groups. The yaks were fed diets with concentrate-to-forage ratios of 50:50 (C50 group), 65:35 (C65 group), or 80:20 (C80 group) (Supplementary Table 1). The 105-day test period comprised a 15-day adaptation period and a 90-day data collection period. The yaks were fed at 8:00 a.m. and 5:00 p.m. daily throughout the whole test period. Feed intake was measured during the pre-feeding period to ensure that there was a surplus of feed before the next day’s feeding. All yaks were allowed to eat and drink freely during the whole test period. The animals in this study were handled in accordance with the Regulations for Laboratory Animals of Beijing. The protocol was approved by the Animal Welfare Committee of China Agricultural University (Permit No. DK1008). The experiments were performed in accordance with the Regulations on the Administration of Laboratory Animal Affairs promulgated by the National Science and Technology Commission of the People’s Republic of China in 1988.

### Ruminal Fluid Sample Collection and Measurement

Before the morning feeding on day 90 of the formal test period, 150 mL of rumen fluid was collected using a transoral rumen cannula sampler. Each sample was filtered through four layers of gauze and dispensed into 15-mL centrifuge tubes, which were stored frozen at −80°C until analysis. The pH of rumen fluid was determined using a Testo 205 portable pH meter (Testo AG, Schwarzwald, Germany). The filtered rumen fluid was centrifuged at 8,000 × *g* at 4°C for 15 min. The supernatant obtained was used to determine the concentrations of volatile fatty acids (VFA) and ammonia nitrogen (NH_3_-N). VFA concentrations were measured by gas chromatography according to the method of Erwin et al. (12). NH_3_-N concentrations were determined by the method of Broderick and Kang (13).

### DNA Extraction, PCR Amplification and Sequencing

Total microbial community DNA was extracted from ruminal fluid samples (15 mL) from all yaks (*n* = 18) using the FastDNA^®^ Spin Kit for Soil (MP Biomedicals, Solon, OH, United States) according to the manufacturer’s instructions. The quality of extracted DNA was determined by 1% agarose gel electrophoresis. DNA concentration and purity were determined using a NanoDrop 2000 spectrophotometer (Thermo Fisher Scientific, Waltham, MA, United States). PCR amplification of the V3–V4 variable region of 16S rRNA genes was performed using primers 338F (5′-ACTCCTACGGGAGGCAGCAG-3′) and 806R (5′-GGACTACHVGGGTWTCTAAT-3′). The amplification procedure comprised predenaturation at 95°C for 3 min, 27 cycles of processing (denaturation at 95°C for 30 s, annealing at 55°C for 30 s, and extension at 72°C for 45 s), stable extension at 72°C for 10 min, and finally storage at 4°C. The PCR instrument was an ABI GeneAmp^®^ Model 9700. The PCR reaction system contained 4 μL of 5 × TransStart FastPfu buffer, 2 μL of 2.5 mM dNTPs, 0.8 μL of upstream primer (5 μM), 0.8 μL of downstream primer (5 μM), 0.4 μL of TransStart FastPfu DNA polymerase, 10 ng of template DNA, with distilled deionized water (ddH_2_O) added to a final volume of 20 μL. PCR reactions were performed in triplicate. PCR products from the same sample were mixed and recovered using 2% agarose gel electrophoresis, purified using the AxyPrep DNA Gel Extraction Kit (Axygen Biosciences, Union City, CA, United States), detected by 2% agarose gel electrophoresis, and quantified using a Quantus™ Fluorometer (Promega, Madison, WI, United States). Purified amplicons were pooled in equimolar amounts and paired-end (PE) sequenced on a MiSeq PE300 platform (Illumina, San Diego, CA, United States) according to standard protocols by Majorbio Bio-Pharm Technology Co., Ltd. (Shanghai, China).

### Sequencing Data Processing and Analysis

The raw sequences were quality-controlled using fastp software (version 0.20.0)^1^ (14) and spliced using FLASH software (version 1.2.7)^2^ (15). In detail, bases were filtered with a quality value < 20 at the ends of reads. A window of 50 bp was set and the back-end bases were truncated from the window if the average quality value within the window was < 20. Reads < 50 bp long after quality control (QC) were filtered and reads containing N bases were removed. Pairs of reads were spliced (merged) into one sequence according to the overlap relationship between PE reads, with a minimum overlap length of 10 bp. The maximum mismatch ratio allowed in the overlap region of the spliced sequence was 0.2, and the non-conforming sequence was screened. Samples were distinguished according to the barcode and primers at the start and end of the sequence. The sequence orientation was adjusted. The number of mismatches allowed for the barcode was 0 and the maximum number of primer mismatches was 2. UPARSE software^3^ (version 7.1) (16) was used to cluster the sequences into operational taxonomic units (OTUs). Chimeras were removed based on 97% similarity (16, 17). Each sequence was annotated for species classification using RDP classifier (18), compared with the Silva 16S rRNA database (v138), with a comparison threshold of 70%.

### Liquid Chromatography-Mass Spectrometry Metabolome Analysis

A 100 μL sample of rumen fluid was added to a 2-mL centrifuge tube. Metabolites were extracted using 400 μL of methanol:water solution (4:1, v/v) with 0.02 mg/mL L-2-chlorophenylalanin as an internal standard. The sample solution was ground in a frozen tissue grinder for 6 min (−10°C, 50 Hz), followed by low-temperature sonication for 30 min (5°C, 40 kHz). After extraction, the samples were left to stand for 30 min (−20°C) and then centrifuged for 15 min (4°C, 13,000 × *g*). The supernatant was pipetted into an injection vial with an internal cannula for analysis. As a part of the system conditioning and QC process, a pooled QC sample was prepared by mixing equal volumes of all samples. The QC sample was tested in the same manner as the analytic samples and was injected at regular intervals (every 10 samples) to monitor the stability of the analysis.

The instrumental platform for LC-MS analysis was an ultra-high performance liquid chromatography tandem time-of-flight mass spectrometry UHPLC-Q Exactive HF-X system from Thermo Corporation (Waltham, MA, United States). In the chromatography, 2 μL of sample was separated on an HSS T3 column (100 mm × 2.1 mm i.d., 1.8 μm) and injected into the mass spectrometer for detection. Mobile phase A was 95% water + 5% acetonitrile (containing 0.1% formic acid). Mobile phase B was 47.5% acetonitrile + 47.5% isopropanol + 5% water (containing 0.1% formic acid). In the separation gradient, from 0 to 3.5 min, mobile phase B was increased from 0 to 24.5% with a flow-rate of 0.40 mL/min; from 3.5 to 5 min, mobile phase B was increased from 24.5 to 65% with a flow-rate of 0.4 mL/min; from 5 to 5.5 min, mobile phase B was increased from 65 to 100% with a flow-rate of 0.4 mL/min; from 5.5 to 7.4 min. mobile phase B was maintained at 100% and the flow-rate was increased from 0.4 to 0.6 mL/min; from 7.4 to 7.6 min, mobile phase B was decreased from 100 to 51.5% and the flow-rate was 0.6 mL/min; from 7.6 to 7.8 min, mobile phase B was decreased from 51.5 to 0% and the flow-rate was decreased from 0.6 to 0.5 mL/min; from 7.8 to 9 min, mobile phase B was maintained at 0% and the flow-rate was decreased from 0.5 to 0.4 mL/min; finally, from 9 to 10 min, mobile phase B was maintained at 0% and the flow-rate was 0.4 mL/min. The column temperature was 40°C. The mass spectrometry conditions were: positive and negative ion scan mode for sample mass spectrometry signal acquisition; mass scan range *m/z*: 70–1,050; ion spray voltage; positive ion voltage, 3,500 V; negative ion voltage, 2,800 V; sheath gas, 40 psi; auxiliary heating gas, 10 psi; ion source heating temperature, 400°C; 20–40–60 V cyclic collision energy; and MS^1^ and MS^2^ resolution 70,000 and 17,500, respectively.

### Metabolomic Data Processing and Analysis

After uploading, the raw LC-MS data were imported into the metabolomics processing software Progenesis QI (Waters Corporation, Milford, MA, United States) for baseline filtering, peak identification, integration, retention time correction, and peak alignment. The resulting data matrix included retention time, mass-to-charge ratio, and peak intensity. The data matrix was retained for at least one set of variables with non-zero values ≥ 80% of the sample, and then vacant values were filled in (the smallest value in the original matrix was used to fill in the vacant values). To reduce the errors caused by sample preparation and instrument instability, the response intensities of the sample mass spectrometry peaks were normalized by the sum normalization method, and the normalized data matrix was obtained. Variables with relative standard deviations (RSDs) > 30% of the QC samples were removed and log_10_ logarithmic processing was performed to obtain the final data matrix for subsequent analysis. The MS and MSMS mass spectrometry information was matched with the metabolic public databases HMDB^4^ and Metlin^5^ to obtain metabolite information. The preprocessed data were uploaded to the Megabio cloud platform^6^ for data analysis. Principal component analysis (PCA) and orthogonal least partial squares discriminant analysis (OPLS-DA) were performed using the R package ropls (version 1.6.2). Stability of the model was evaluated using seven round-robin interaction validation, with *P* < 0.05 considered to indicate statistical significance. In addition, Student’s *t*-test analysis was performed. The selection of significantly differentially expressed metabolites (DEMs) was performed based on the variable weight value (VIP) obtained from the OPLS-DA model and the *P*-value from Student’s *t*-test, with metabolites with VIP > 1 and P < 0.05 being significantly DEMs. A total of 1,453 differential metabolites were screened, and metabolic pathway annotation was performed using the Kyoto Encyclopedia of Genes and Genomes (KEGG) database^7^ to identify the pathways in which DEMs are involved. Pathway enrichment analysis was performed using the Python package scipy.stats (version 1.0.0), and the most relevant biological pathways to the experimental treatments were identified by Fisher’s exact test. Correlations between different metabolites and bacterial communities were assessed by Spearman correlation analysis using the Python package scipy.stats (version 1.0.0). *P*-values were adjusted with false discovery rate and corrected *P*-values < 0.05 were considered statistically significant.

## Results

### Rumen Fermentation Parameters

Table 1 presents the effect of dietary concentrate-to-forage ratio on rumen fermentation parameters (pH, NH_3_-N, VFA). Ruminal pH was lower in the C80 group than in the other two groups. The NH_3_-N content of the C50 group was significantly lower than that of the other two groups. Total VFA, acetate, propionate, and isobutyrate concentrations were higher in the C80 group than in the other two groups (*P* < 0.05). The butyrate concentration was lowest in the C50 group, while the acetate to propionate ratio was higher in the C50 group than in the other two groups (*P* < 0.05). No significant differences were observed in the contents of isovalerate and valerate among the three treatment groups.

**TABLE 1:** Rumen fermentation parameters affected by dietary concentrate-to-forage ratios.

Item	C50	C65	C80	SEM	*P*-value
pH	6.75*^a^*	6.49*^ab^*	6.25*^b^*	0.072	0.0108
NH_3_-N, mg/100 mL	9.81*^b^*	12.51*^a^*	12.61*^a^*	0.491	0.0208
Total VFA, mmol/L	78.35*^b^*	82.86*^b^*	90.11*^a^*	1.751	0.0113
VFA, mmol/L					
Acetate	51.93*^a^*	47.78*^b^*	46.59*^b^*	0.931	0.0369
Propionate	10.29*^c^*	14.22*^b^*	22.48*^a^*	1.364	<0.0001
Isobutyrate	0.69*^b^*	1.02*^a^*	1.10*^a^*	0.059	0.0041
Butyrate	10.95*^b^*	15.11*^a^*	14.96*^a^*	0.543	<0.0001
Isovalerate	3.15	3.18	3.36	0.086	0.5869
Valerate	1.34	1.55	1.62	0.096	0.4746
Acetate:Propionate	5.60*^a^*	3.41*^b^*	2.07*^c^*	0.418	<0.0001

*C50, dietary concentrate-to-forage ratio 50:50; C65, dietary concentrate-to-forage ratio 65:35; C80, dietary concentrate-to-forage ratio 80:20; SEM, standard error of the mean; VFA, volatile fatty acid.*

*^a,b,c^Indicate significant differences (P ≤ 0.05).*

### Rumen Bacterial Abundance and Diversity

A total of 1,144,370 sequences were obtained after 16S rRNA high-throughput sequencing of 18 rumen fluid samples from yaks in the three treatment groups. Among them, 421,181 sequences were obtained from group C50; 430,450 sequences from group C65; and 292,739 sequences from group C80. The average sequence length was 416 bp, and a total of 2,324 OTUs were identified (97% sequence similarity level). Of these, 1,495 OTUs were common to the three treatment groups (Supplementary Figure 1). The OTUs in each group were used to generate rarefaction curves, which were used to assess whether the sequencing depth was sufficient (Supplementary Figure 2). As the number of sample reads increased, the identification rate of OTUs gradually decreased and then plateaued, indicating that the sequencing depth was sufficient to assess the major members of the rumen bacterial community.

**FIGURE 1:**
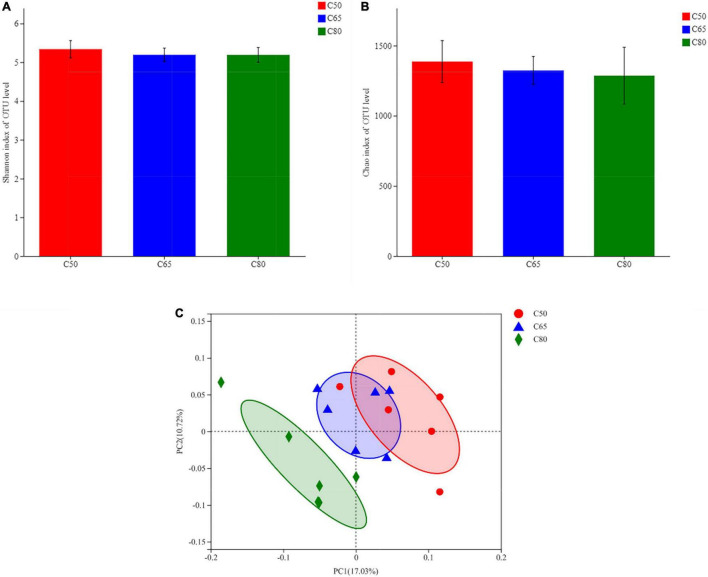
Diversity and richness of bacterial communities. **(A,B)** Alpha diversity metrics. **(A)** Shannon and **(B)** Chao 1 indexes for rumen microbiota. **(C)** Plot of principal coordinate analysis. A shorter distance between the sample points denotes greater similarity of the bacteria. C50, dietary concentrate-to-forage ratio 50:50; C65, dietary concentrate-to-forage ratio 65:35; C80, dietary concentrate-to-forage ratio 80:20.

Figure 1 depicts the alpha diversity indices of the rumen bacterial communities, including the Shannon and Chao1 indices (Figures 1A,B). There was no significant difference in the alpha diversity indices of the three groups. The beta diversity of the rumen bacterial community was used to study the degree of similarity in the composition of the sampled communities. Principal coordinate analysis (PCoA) showed a clear distinction between the C80 group and the groups C50 and C65 (Figure 1C).

### Ruminal Bacterial Community Composition and Species Differences

**FIGURE 2:**
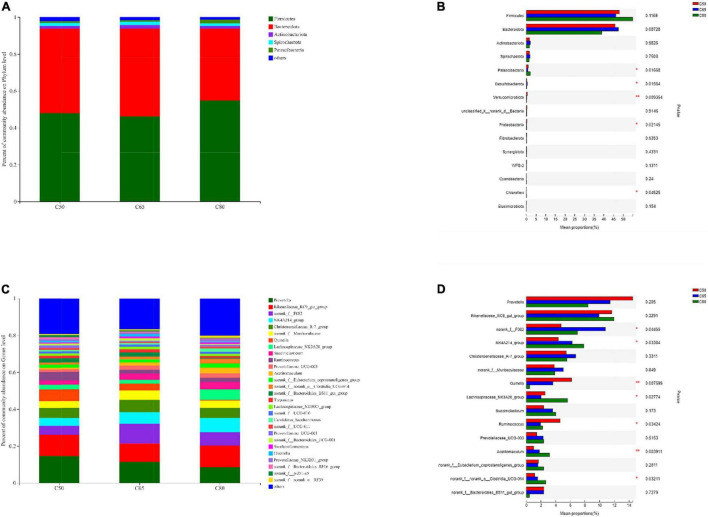
Relative abundance of rumen bacteria at the **(A,B)** phylum and **(C,D)** genus levels in the three groups. C50, dietary concentrate-to-forage ratio 50:50; C65, dietary concentrate-to-forage ratio 65:35; C80, dietary concentrate-to-forage ratio 80:20; *0.01 < *P* ≤ 0.05; **0.001 < *P* ≤ 0.01.

Figure 2 depicts the composition of the rumen bacterial communities and details of the intergroup differences in the top 15 bacterial phyla and genera in terms of abundance. At the phylum level, the rumen bacterial community consisted mainly of *Firmicutes* and *Bacteroidota* (Figure 2A), which were not affected by changes in the dietary concentrate-to-forage ratio. However, the relative abundances of *Patescibacteria*, *Desulfobacterota*, *Verrucomicrobiota*, *Proteobacteria*, and *Chloroflexi* were significantly different (*P* < 0.05) dependent on the dietary concentrate-to-forage ratio (Figure 2B). At the genus level, the relative abundance of *norank_f_F082*, *NK4A214_group*, *Lachnospiraceae_NK3A20_group*, *Acetitomaculum*, and *norank_f_norank_o_Clostridia_UCG-014* increased when the dietary concentrate-to-forage ratio significantly increased (*P* < 0.05), while *Quinella* and *Ruminococcus* showed the opposite result (*P* < 0.05). Among them, the abundance of *norank_f_F082* in the C65 group was higher than that in C50 (*P* < 0.05). The relative abundance of *NK4A214_group*, *Lachnospiraceae_NK3A20_group*, *Acetitomaculum*, and *norank_f_norank_o_Clostridia_UCG-014* in the C80 group was the highest among the three groups. The abundance of *NK4A214_group* and *norank_f_norank_o_Clostridia_UCG-014* was higher in the C80 group than in the C50 group (*P* < 0.05), while the abundance of *Lachnospiraceae_NK3A20_group* and *Acetitomaculum* was higher in the C80 group than in the C50 and C65 groups (*P* < 0.05). In the C50 group, the abundance of *Quinella* was higher than that in the C80 group (*P* < 0.01), and the abundance of *Ruminococcus* was higher than that in the C65 group (*P* < 0.05) (Figure 2D).

### Correlation of Rumen Bacteria With Rumen Fermentation Parameters

**FIGURE 3:**
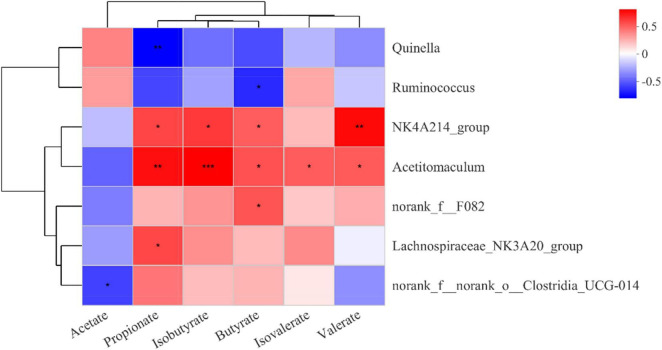
Correlation of rumen bacteria with rumen fermentation parameters. Each row in the figure represents a genus, each column represents a metabolite, and each cell represents a Pearson correlation coefficient between a genus and a metabolite. Red denotes positive correlation and blue denotes negative correlation; *0.01 < *P* ≤ 0.05; **0.001 < *P* ≤ 0.01; ***P ≤ 0.001.

Based on Pearson correlation coefficients, genera with significant differences in the top 15 abundance rankings were significantly correlated with rumen fermentation parameters (Figure 3). *NK4A214_group* and *Acetitomaculum* were positively correlated with the abundance of propionate, isobutyrate, and butyrate. *Lachnospiraceae_NK3A20_group* was positively correlated with propionate, while *Quinella* was negatively correlated with propionate. *Norank_f__norank_o__Clostridia_UCG-014* was negatively correlated with acetate.

### Ruminal Metabolite Identification and Differential Metabolite Analysis

**FIGURE 4:**
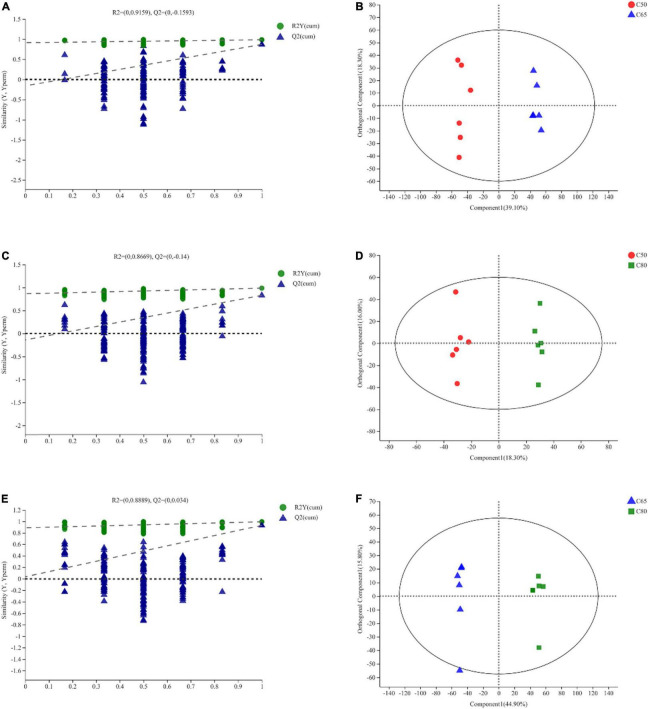
Orthogonal partial least squares discriminant analysis (OPLS-DA) of yak rumen metabolites. OPLS-DA permutation test. The abscissa represents the permutation retention of the permutation test. The ordinate represents the value of the R2 (green circle) and Q2 (blue triangle) permutation tests. The two dashed lines represent the regression lines of R2 and Q2, respectively **(A,C,E)**. The OPLS-DA score map, through orthogonal rotation, filters out information irrelevant to the group, so that the difference between the groups can be better distinguished and the performance of the model can be improved. The abscissa represents the degree of interpretation of the first predicted principal component. The ordinate represents the degree of interpretation of the first orthogonal component **(B,D,F)**. C50, dietary concentrate-to-forage ratio 50:50; C65, dietary concentrate-to-forage ratio 65:35; C80, dietary concentrate-to-forage ratio 80:20.

PCA score plots for the three treatment groups (Supplementary Figure 3) showed good aggregation of the samples within groups and clear separation between groups, confirming the validity of the experimental data. Figure 4 depicts the OPLS-DA score plot, which showed a clear separation between the three treatment groups. All samples were within the 95% Hotelling T2 ellipse, except for one rumen fluid sample that was located outside the ellipse (Figures 4B,D,F). The results of permutation tests between the groups (C50 vs. C65, R2Y = 0.9159, Q2Y = −0.1593 < 0; C50 vs. C80, R2Y = 0.8669, Q2Y = −0.14 < 0; C65 vs. C80, R2Y = 0.8889, Q2Y = 0.034) indicated that the model was stable, reliable and valid (Figures 4A,C,E).

**FIGURE 5:**
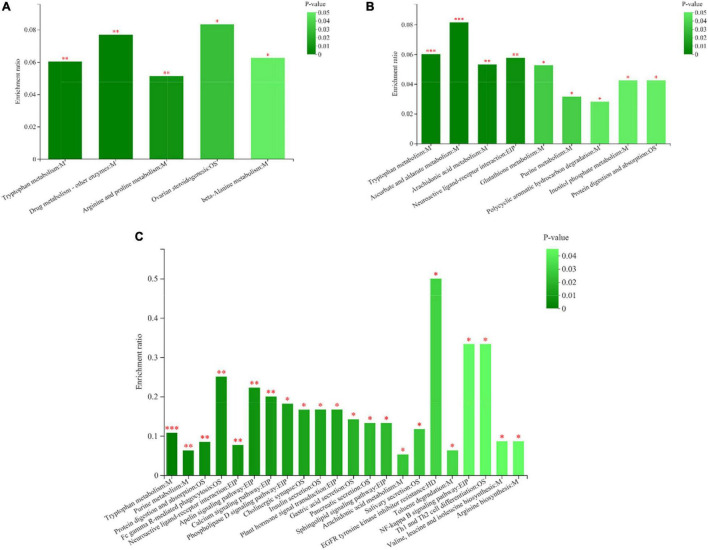
Metabolic pathway enrichment analysis. **(A)** Pathway enrichment map of metabolites between yaks in groups C50 and C65. **(B)** Pathway enrichment map of metabolites between yaks in groups C50 and C80. **(C)** Pathway enrichment map of metabolites in yaks between groups C65 and C80. The abscissa indicates the pathway name. The ordinate represents the enrichment. The color gradient of the columns indicates the significance of the enrichment; the darker the color, the more significant the enrichment of the KEGG term. CP, EIP, GIP, HD, M, and OS are the class names of metabolic pathways in the KEGG annotation. CP, Cellular Processes; EIP, Environmental Information Processing; GIP, Genetic Information Processing; HD, Human Diseases; M, Metabolism; OS, Organismal Systems. C50, dietary concentrate-to-forage ratio 50:50; C65, dietary concentrate-to-forage ratio 65:35; C80, dietary concentrate-to-forage ratio 80:20. *0.01 < *P* ≤ 0.05; ^**^0.001 < *P* ≤ 0.01; ^***^P ≤ 0.001.

A total of 1,453 metabolites were identified in the three groups of rumen fluid samples by LC-MS analysis and screened using the VIP > 1, *P* < 0.05 criterion. Hence, 499 DEMs were detected in group C50 vs. C65, 284 DEMs in group C50 vs. C80, and 592 DEMs in group C65 vs. C80 (Supplementary Table 2). These different metabolites were mainly lipids and lipid-like molecules, phenylpropanoids and polyketides, organic heterocyclic compounds, organic acids and derivatives, organic oxygen compounds, benzenoids, nucleosides, nucleotides, and analogs. Table 2 lists the main differential metabolites between the three treatment groups. Compared with the C50 group, 3-methylindole, pantothenic acid, D-pantothenic acid, and 20-hydroxy-leukotriene E4 were downregulated in the C65 group, while spermine and ribose 1-phosphate were upregulated. Compared to the C50 group, Xanthurenic acid, tyramine, ascorbic acid, D-glucuronic acid, 6-keto-prostaglandin F1a, lipoxin B4, and deoxyadenosine monophosphate were upregulated in the C80 group, while 3-methylindole and 20-hydroxy-leukotriene E4 were downregulated. All metabolites were upregulated in the C80 group compared with the C65 group (Table 2). KEGG pathway enrichment analysis showed that the number of differential metabolic pathways increased with the increase of the dietary concentrate-to-forage ratio (Figure 5). For group C50 vs. C65, the differential metabolic pathways were mainly related to amino acid metabolism (tryptophan metabolism, arginine and proline metabolism, beta-alanine metabolism) (Figure 5A). In contrast, for group C50 vs. C80, there were significant differences in metabolism of tryptophan, ascorbate and aldarate, arachidonic acid, glutathione, and purine, protein digestion and absorption, and neuroactive ligand-receptor interaction (Figure 5B). In group C65 vs. C80, the differential metabolic pathways were mainly related to tryptophan metabolism, purine metabolism, arachidonic acid metabolism, amino acid biosynthesis (arginine, valine, leucine, and isoleucine), protein digestion and absorption, and neuroactive ligand-receptor interaction (Figure 5C).

**TABLE 2:** Main differential metabolites involved in different metabolic pathways between the three groups.

Groups*^a^*	Metabolite	VIP*^b^*	Fold-change	*P*-value	Type
**C50 vs. C65**					
	3-Methylindole	1.66472	0.858	0.002	Down
	Spermine	2.08196	1.243	0.014	Up
	Pantothenic Acid	1.38026	0.921	0.007	Down
	D-Pantothenic acid	1.26570	0.931	<0.001	Down
	20-Hydroxy-leukotriene E4	2.67009	0.675	<0.001	Down
	Ribose 1-phosphate	1.34407	1.093	0.033	Up
**C50 vs. C80**					
	3-Methylindole	2.35974	0.888	0.003	Down
	Xanthurenic acid	1.81221	1.080	0.004	Up
	Tyramine	1.93671	1.088	0.003	Up
	Ascorbic Acid	1.48855	1.066	0.028	Up
	D-Glucuronic acid	1.77684	1.072	0.006	Up
	20-Hydroxy-leukotriene E4	3.65462	0.778	<0.001	Down
	6-Keto-prostaglandin F1a	1.16738	1.027	0.021	Up
	Lipoxin B4	3.18257	1.468	0.027	Up
	Deoxyadenosine monophosphate	2.13436	1.074	0.015	Up
**C65 vs. C80**					
	Xanthurenic acid	1.10104	1.083	0.007	Up
	L-Valine	1.13278	1.049	<0.001	Up
	*N*-Acetyl-L-glutamate 5-semialdehyde	1.29736	1.081	<0.001	Up
	*N*-Acetyl-L-glutamic acid	1.00194	1.050	0.002	Up
	Tyramine	1.46955	1.127	0.001	Up
	6-Keto-prostaglandin F1a	1.32049	1.064	0.000	Up
	Lipoxin B4	2.14669	1.597	0.015	Up
	Xanthosine	1.09312	1.067	0.004	Up
	Thymine	1.87636	1.145	<0.001	Up
	Deoxyinosine	1.93112	1.149	<0.001	Up
	Uric acid	1.21992	1.065	0.001	Up

*^a^C50, dietary concentrate-to-forage ratio 50:50; C65, dietary concentrate-to-forage ratio 65:35; C80, dietary concentrate-to-forage ratio 80:20. ^b^VIP, variable importance in the projection. All differential metabolites listed here had values VIP > 1 and P < 0.05.*

### Relationships Between Metabolites and Rumen Bacteria

**FIGURE 6:**
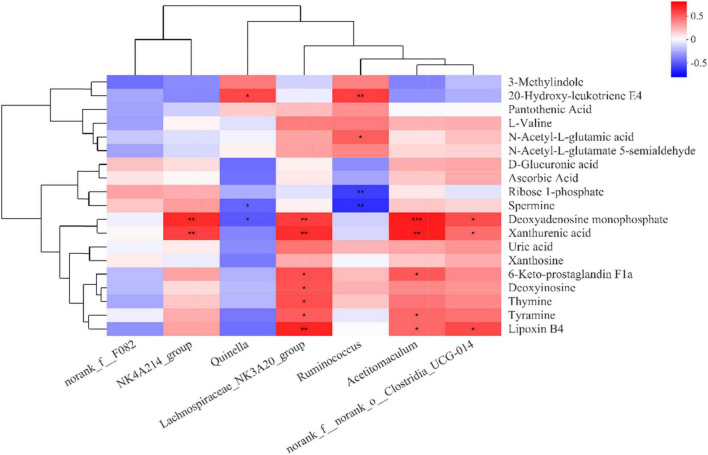
Correlation matrix between rumen bacterial genera and rumen differential metabolites (derived from Table 2). Each column in the graph represents a genus, each row represents a metabolite, and each cell represents a Pearson correlation coefficient between a genus and a metabolite. Red denotes positive correlation and blue denotes negative correlation. *0.01 < *P* ≤ 0.05; **0.001 < *P* ≤ 0.01; ***P ≤ 0.001.

Changes in rumen metabolites reflect the dynamics of the rumen microbial community. Correlation analysis using microbial communities with rumen metabolites contributes to a comprehensive understanding of rumen microbial community composition and function. Therefore, we correlated seven differential bacterial genera in the top 15 abundance rankings with key differential metabolites in the metabolic pathways (Figure 6). The abundance of *NK4A214_group*, *Lachnospiraceae_NK3A20_group*, *Acetitomaculum*, and *norank_f_norank_o_Clostridia_UCG-014* all increased with the increase of dietary concentrate-to-forage ratio, and they all showed a strong positive correlation with metabolites. Deoxyadenosine monophosphate and xanthurenic acid were all positively correlated with the abundances of *NK4A214_group*, *Lachnospiraceae_NK3A20_group*, *Acetitomaculum*, and *norank_f__norank_o__Clostridia_UCG-014*, while deoxyadenosine monophosphate was negatively correlated with *Quinella*. Both deoxyinosine and thymine were positively correlated with *Lachnospiraceae_NK3A20_group*. *Lachnospiraceae_NK3A20_group* and *Acetitomaculum* were positively correlated with lipoxin B4, 6-keto-prostaglandin F1a, and tyramine. Lipoxin B4 was also positively correlated with *norank_f__norank_o__Clostridia_UCG-014*. Spermine, which, like tyramine, is a biogenic amine, was negatively correlated with *Quinella* and *Ruminococcus*, while 20-hydroxy-leukotriene E4 was positively correlated with *Quinella* and *Ruminococcus*. *Ruminococcus* was also negatively correlated with ribose 1-phosphate and positively correlated with *N*-acetyl-L-glutamic acid.

## Discussion

The symbiotic relationship between animals, microbiota, and feed is essential for healthy yak breeding as well as for efficient use of feed. In this study, we investigated the effect of different dietary concentrate-to-forage ratios on the rumen microbiota and metabolome of yaks. The study found that rumen pH decreased with a higher percentage of concentrate, which may be caused by increased starch intake. Different dietary composition may lead to differences in the internal rumen environment. A higher percentage of concentrate in the diet will increase fermentable carbohydrates in the rumen, which will lead to a decrease in rumen pH, along with an increase in NH_3_-N and VFA in the rumen. The C80 group had the highest NH_3_-N content among the three groups, which could be explained by the high-concentrate diet producing more rumen microbial protein. In this study, increasing the proportion of dietary concentrate increased the total VFA yield and the yields of propionate, butyrate and isobutyrate, but decreased the amount of acetate and the acetate:propionate ratio, which was essentially consistent with the results of other studies (10, 19, 20). Our findings confirm that feeding a high-concentrate diet shifted the rumen fermentation pattern of yaks from acetate to propionate. A decreased acetate:propionate ratio can reflect an increase in feed energy use efficiency (8). Although this study did not include production performance, changes in the acetate:propionate ratio can provide guidance for future production. Concentrates contain a higher proportion of fermentable carbohydrates, and feeding a diet with a higher proportion of concentrates will lead to an increase in the VFA profiles of yaks. VFAs are the major products of rumen microbial fermentation, and the molar ratio of individual VFAs in the rumen may indicate changes in the bacterial community composition (21). We believe that changes in the dietary concentrate-to-forage ratio will not only alter the available fermentation substrates, but also the rumen environment, which will affect the metabolic pathways used by microorganisms (22). Therefore, changes in bacterial communities in the yak rumen might be one of the reasons for the changes in the abundances of VFAs.

The present study describes the composition of the yak rumen bacterial community. Consistent with previous studies (8, 23, 24), we found that the dominant phyla in the rumen of yak were *Firmicutes*, *Bacteroidota*, and *Actinobacteriota*. Among them, *Firmicutes* and *Bacteroidota* account for approximately 90% of all the bacterial phyla. Previous studies have reported the ability of *Firmicutes* and *Bacteroidota* to degrade structural polysaccharides in the rumen (25). However, the abundances of *Firmicutes* and *Bacteroidota* were not affected by changes in the dietary concentration ratio, possibly because of the ability of rumen microorganisms to adapt to changing levels of dietary concentrates through self-regulation. Again, this could explain the lack of significant differences in alpha diversity indicators (Shannon and Chao1 indices) among the three groups. Although the increase in the ratio of concentrate-to-forage had no effect on bacterial diversity and richness in the samples, it significantly affected the beta-diversity between samples and the differences in community composition between treatment groups. PCoA plots revealed clear clustering and clearly distinguished between the three groups. At the genus level, we detected seven differential genera (among the top 15 in abundance). Among them, *NK4A214_group*, *Lachnospiraceae_NK3A20_group*, *Acetitomaculum*, and *norank_f_norank_o_ Clostridia_UCG-014* increased with the increase of concentrate-to-forage ratio. A study by Liu et al. (10) reported a higher abundance of *NK4A214_group* in the rumen of concentrate-fed yaks, which is consistent with the results of this study. The *NK4A214_group* belongs to the *Ruminococcus* family, and this change may be related to its ability to digest resistant starch (26, 27), because diets with high-concentrate ratios feature more resistant starch. This can also explain the positive correlation between the abundances of the *NK4A214_group* and propionate, butyrate, and isobutyrate. *Acetitomaculum* was also positively correlated with propionate, butyrate, and isobutyrate. *Acetitomaculum* is closely related to the production of propionate (28), which may be caused by the increase of total fermentable carbohydrates in the feed. Propionate was also positively correlated with the *Lachnospiraceae_NK3A20_group*. This group ferments glucose to produce lactic acid (29). The *Lachnospiraceae_NK3A20_group* was most abundant in the C80 group, and the starch content in the C80 group was also higher, which may be the reason for the positive correlation of this group with propionate. In other words, an increase in dietary fermentable carbohydrates favored the growth of *Lachnospiraceae_NK3A20_group*. The negative correlation of *norank_f_norank_o_ Clostridia_UCG-014* with acetate was validated by its concentration in the rumen. We also found that the abundance of *Quinella* decreased with increasing concentrate ratio, echoing its negative correlation with propionate, suggesting that *Quinella* growth may be limited by rumen pH or propionate concentration. *Ruminococcus* showed the same trend. A previous study reported that *Ruminococcus* are the main cellulolytic bacteria in the rumen (30). The increase in the concentrate ratio resulted in a decrease in crude fiber in the diet, which may be the reason for the decrease in the abundance of *Ruminococcus* with the increase in the concentrate ratio. Notably, in addition to these changes in taxonomically classified bacteria, unclassified *norank_f__F082* was also significantly affected by dietary treatment. The exact role of *norank_f__F082* is unclear, but its abundance increases with the proportion of concentrate, suggesting that it may play an important role in the digestion of non-structural carbohydrates.

In the present study, the tryptophan metabolic pathway was significantly enriched in all three treatment groups. Tryptophan is an essential aromatic amino acid, and among the 20 common typical amino acids, tryptophan has the highest molecular weight (31). Although tryptophan is the least abundant amino acid in proteins and cells, it is a biosynthetic precursor for large number of microbial and host metabolites (32, 33). A 3-Methylindole and xanthurenic acid are metabolites of tryptophan, and the former was downregulated in groups C65 and C80 compared to the C50 group. Compared with groups C50 and C65, xanthuronic acid was upregulated in group C80. The possible explanation for this phenomenon is that the increase of the concentrate ratio leads to increased generation of xanthurenic acid from tryptophan (31, 34). In this way, subsequent reference numbers will be added by one digit, so that the sequence will not be disordered. Previous studies have shown that the degradation of tryptophan in animals is related to the vitamin B_6_ family, and that lack of vitamin B_6_ in the body results in the metabolism of tryptophan to xanthurenic acid (35, 36). The high proportion of grains contained in the C80 diets resulted in a large amount of fermentable carbohydrate. The “pyridoxal phosphate” coenzyme produced by vitamin B_6_ is prominent in catalysis of the conversion of glucose to glucose 6-phosphate (37, 38). Vitamin B_6_ will participate in the metabolism of carbohydrates in yaks. In turn, tryptophan will tend to be metabolized to xanthurenic acid. This change in xanthurenic acid also appears to be associated with microbes in the rumen. We found that *NK4A214_group*, *Lachnospiraceae_NK3A20_group*, *Acetitomaculum*, and *norank_f_norank_o_Clostridia_UCG-014* were positively correlated with xanthurenic acid. The functions of these four bacterial genera are related to fibrinolysis and starch degradation. Thus, bacterial degradation of carbohydrates is likely the main reason for the changes in tryptophan metabolic pathways.

L-valine, *N*-acetyl-L-glutamate-5-semialdehyde and *N*-acetyl-L-glutamic acid were upregulated in the C80 group compared with the C65 group. The latter two metabolites participate in the arginine biosynthesis pathway as precursors for the synthesis of arginine (39). Previous studies have reported that rumen microorganisms can synthesize amino acids *de novo* using VFA or other substances as carbon sources, and nitrogen compounds, such as ammonia, as nitrogen sources (40, 41). In the present study, higher concentrations of propionate and butyrate likely provided more substrates for amino acid synthesis in the C80 group at lower rumen pH. Therefore, this evidence suggests that the C80 group has stronger rumen amino acid metabolism compared to the C50 and C65 groups.

Biogenic amines (spermine and tyramine) are normal physiological components in animals, plants, and most microorganisms, and have important physiological functions in cells (42, 43). In our study, elevated spermine levels were observed in the C65 group compared to the C50 group, as were elevated tyramine levels in the C80 group compared to the C50 and C65 groups. This is consistent with previous research findings (44, 45). Relevant studies have confirmed that the increase of biogenic amines in animals is closely related to ruminal acidosis (46, 47), which suggests that our diet with a high proportion of concentrate may cause the occurrence of rumen acidosis in yak, and corresponding measures should be taken in production to ensure the health of the animals. Interestingly, D-Glucuronic acid and Ascorbic Acid, which are involved in the secondary glucose degradation pathway (glucuronide pathway), were identified in our study. In the rumen, glucose-1-phosphate from feed degradation reacts with UTP to generate D-glucuronic acid after dehydrogenation and hydrolysis (48). D-Glucuronic acid generates ascorbic acid under the action of NADPH and related enzymes (49). In the C80 group, the levels of D-Glucuronic acid and Ascorbic Acid were elevated, indicating that the increase in the proportion of feed concentrate enhanced the metabolism of carbohydrates in the rumen. Pantothenic acid is an important part of coenzyme A, which is a key coenzyme in the metabolism of fatty acids and pyruvate in animals (50–52). Compared with group C50, pantothenic acid and D-pantothenic acid were downregulated in group C65, which may be due to the enhanced metabolism of fatty acid and pyruvate in yak due to the increase in the proportion of concentrate.

Changes in the concentrations of three lipid-like molecules (lipoxin B4, 6-keto-prostaglandin F1a, and 20-hydroxy-leukotriene E4) in the rumen indicated that changes in the ratio of concentrate-to-forage had an effect on the biosynthesis and metabolism of fatty acids. All three of these metabolites are involved in the metabolism of arachidonic acid. Arachidonic acid is a ubiquitous component in every mammalian cell. This acid is an unsaturated fatty acid that is important for normal cell membrane fluidity. It also acts as a mediator of bioactive lipid mediators such as prostaglandins and leukotrienes (53, 54). Compared with C50, 20-hydroxy-leukotriene E4 was downregulated in groups C65 and C80, whereas 6-keto-prostaglandin F1a and lipoxin B4 were upregulated in both groups C65 and C80. In the metabolism of arachidonic acid, 6-keto-prostaglandin F1a belongs to the cyclooxygenase pathway (55), while 20-hydroxy-leukotriene E4 and lipoxin B4 belong to the lipoxygenase pathway (54). 6-Keto-prostaglandin F1a and lipoxin B4 both play roles in regulating immune response (56, 57). Therefore, we speculate that the upregulation of 6-keto-prostaglandin F1a and lipoxin B4 may be due to the decrease of rumen pH caused by the increase of the proportion of concentrate in the feed, which leads to damage to the rumen epithelium. This will activate immune response in the yak, with the preferential metabolism of arachidonic acid to 6-keto-prostaglandin F1a and lipoxin B4, which have immunomodulatory effects. Moreover, 6-keto-prostaglandin F1a and lipoxin B4 were both positively correlated with *Lachnospiraceae_NK3A20_group* and *Acetitomaculum*, and these two bacterial genera were also positively correlated with propionic acid. These findings suggest that arachidonic acid metabolism may be related to changes in the acidity of the rumen. Similar conclusions were drawn in the study by Mu et al. (58), confirming that the acidity of the rumen may be responsible for changes in arachidonic acid metabolites such as prostaglandins. Interestingly, arachidonic acid also serves as a specific and sensitive plasma biomarker for average daily gain in bulls (59). The above evidence suggests that elevated dietary concentrate levels lead to enhanced arachidonic acid metabolism, which may serve as a characteristic metabolic pathway for high-concentrate feeding patterns.

Notably, changes in the concentrations of several metabolites (xanthosine, deoxyinosine, deoxyadenosine monophosphate, and uric acid) related to purine metabolism were observed in this study. Nitrogen from feed is degraded in the rumen by the microbiota and reused for microbial nucleic acid synthesis (10). These nucleic acids are first degraded into purine nucleosides (e.g., xanthosine and deoxyinosine) through *de novo* synthesis and salvage pathways, then enzymatically degraded into purine bases. Finally, purine degradation products are formed (60). Previous studies have demonstrated that high grain feeding increases rumen bacterial degradation products, such as xanthine and thymine (41, 46). Among them, xanthine is considered a biomarker of microbial protein synthesis (10, 61, 62). Higher levels of anthosine (the precursor of xanthine) and thymine were also found in our study in the C80 group. This may be due to the increase in the proportion of concentrate, which leads to the degradation of more bacterial nucleic acids. In addition, deoxyadenosine monophosphate and deoxyinosine were positively correlated with bacterial genera (*NK4A214_group*, *Lachnospiraceae_NK3A20_group*, *Acetitomaculum*, and *norank_f_norank_o_Clostridia_UCG-014*) with increased abundance in the rumen, and were negatively correlated with genera with decreased abundance (*Quinella*). This association of purine metabolites with microorganisms also confirmed the above speculation. The findings suggest that an increased concentrate ratio contributes to purine metabolism in the rumen.

There is increasing evidence that animal phenotypic characteristics are closely related to rumen microbes, and that the concentrations of metabolites in the rumen affect rumen bacterial function (10, 11, 63). Overall, the relative changes of rumen microbes and metabolites and their associations may be the main factors affecting yak production performance. Here, combined rumen microbiome and metabolomics analyses clarified associations between bacterial genera and metabolites that were significantly affected by the feed-to-concentrate ratio. The findings provide a more comprehensive understanding of the complex relationships between diet and rumen microbial community function. The findings also provide a rationale for developing appropriate feeding strategies for yak. As a limitation, this study did not address the changes of metabolites after they enter the blood. Understanding the relationships between the rumen metabolome and the serum metabolome and the mechanisms of the interaction between the two and rumen microbes deserves further study.

## Data Availability Statement

All obtained raw sequence datasets have been uploaded to the NCBI Sequence Read Archive (SRA) with the accession number PRJNA820285. This data can be found here: https://www.ncbi.nlm.nih.gov/bioproject/PRJNA820285.

## Ethics Statement

The animal study was reviewed and approved by the Animal Welfare Committee of China Agricultural University. Written informed consent was obtained from the owners for the participation of their animals in this study.

## Author Contributions

SL, SC, QM, and ZZ designed the study. SY, DD, and HW performed the sample process. SY performed the data analysis and wrote the manuscript. HW and ZZ revised the manuscript. All authors have read and approved the final manuscript.

## Conflict of Interest

The authors declare that the research was conducted in the absence of any commercial or financial relationships that could be construed as a potential conflict of interest.

## Publisher’s Note

All claims expressed in this article are solely those of the authors and do not necessarily represent those of their affiliated organizations, or those of the publisher, the editors and the reviewers. Any product that may be evaluated in this article, or claim that may be made by its manufacturer, is not guaranteed or endorsed by the publisher.

## Footnotes

1
https://github.com/OpenGene/fastp

2
http://www.cbcb.umd.edu/software/flash

3
http://drive5.com/uparse/

4
http://www.hmdb.ca/

5
https://metlin.scripps.edu/

6
https://cloud.majorbio.com

7
https://www.kegg.jp/kegg/pathway.html

## Supplementary Material

Supplementary Figure 1 Rarefaction curves for each sample in three groups.

Supplementary Figure 2 Venn diagram of Operational Taxonomic Units (OTUs) in three groups.

Supplementary Figure 3 Principal component analysis (PCA) score plots of metabolite profile.

Supplementary Table 1 Composition and nutrient levels of experimental diets.

Supplementary Table 2 List of identified metabolites.
