# Supplementary figures and images for: Dietary Concentrate-to-Forage Ratio Affects Rumen Bacterial Community Composition and Metabolome of Yaks
Source: Front Nutr. 2022 Jul 14;9:927206. doi: 10.3389/fnut.2022.927206 (PMC9329686; doi:10.3389/fnut.2022.927206)

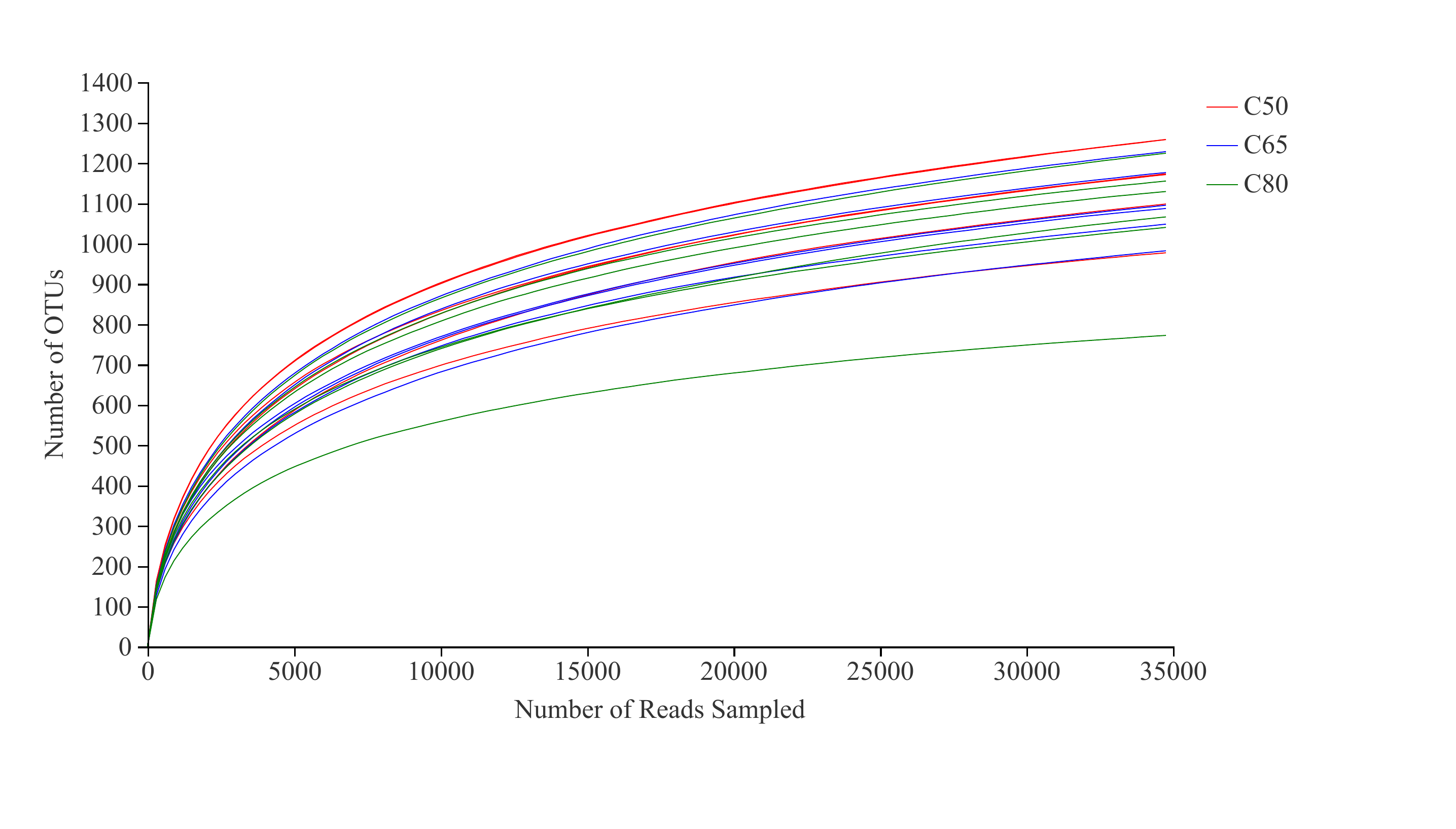

Supplement: Supplementary Figure 1 — Rarefaction curves for each sample in three groups. [file Image_1.PNG]

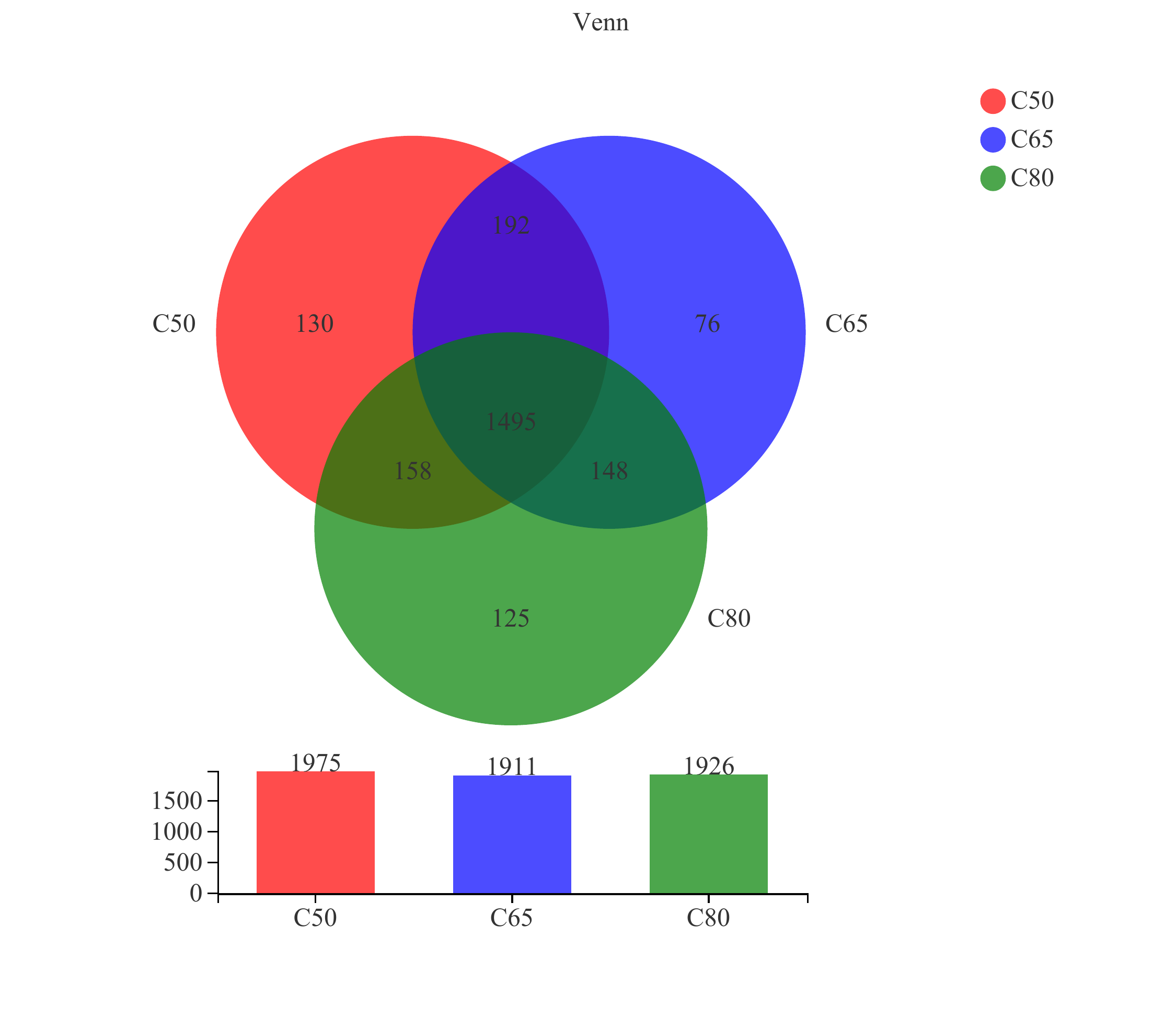

Supplement: Supplementary Figure 2 — Venn diagram of Operational Taxonomic Units (OTUs) in three groups. [file Image_2.PNG]

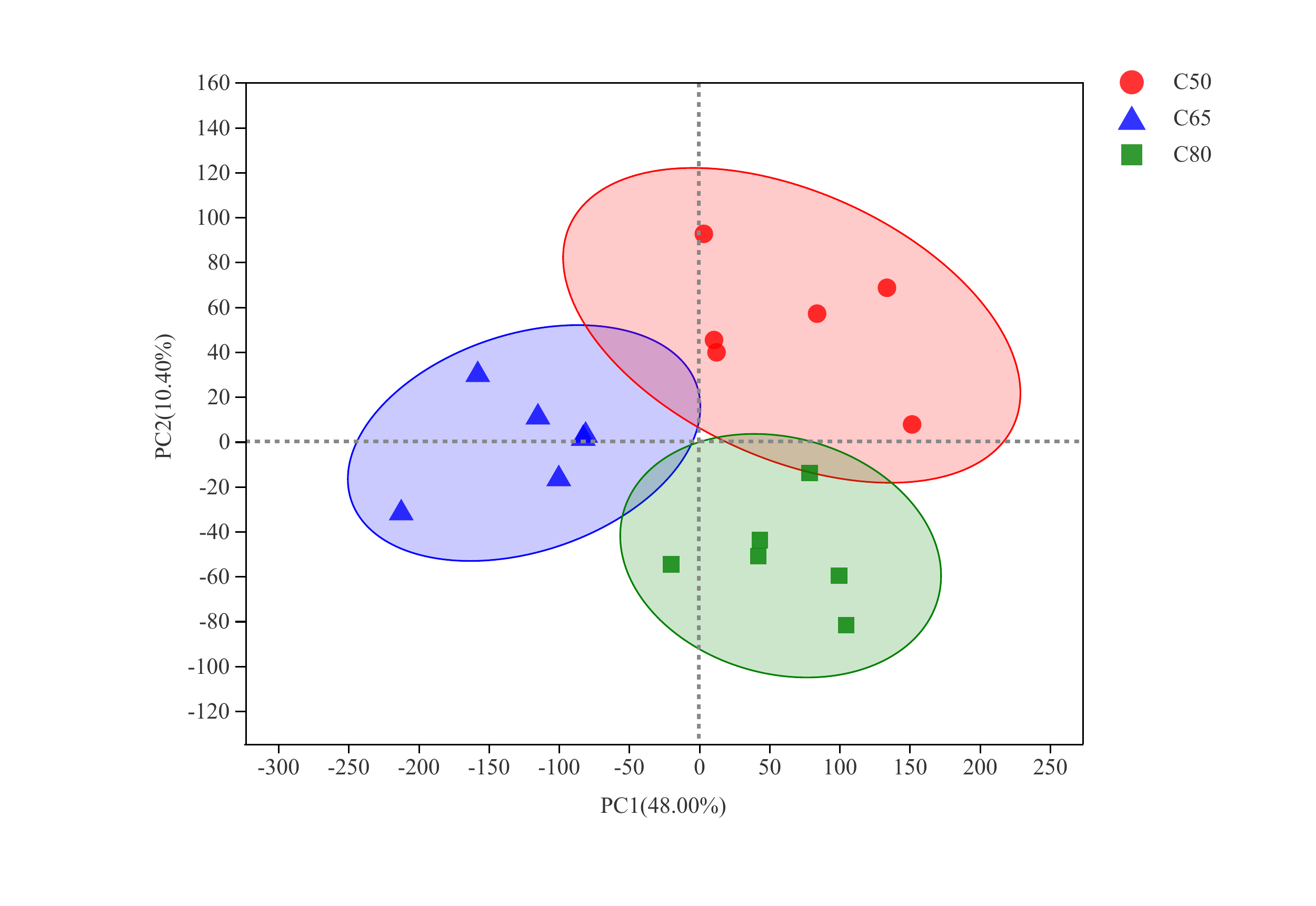

Supplement: Supplementary Figure 3 — Principal component analysis (PCA) score plots of metabolite profile. [file Image_3.PNG]
