# Supplementary material for: Dietary Concentrate-to-Forage Ratio Affects Rumen Bacterial Community Composition and Metabolome of Yaks
Source: Front Nutr. 2022 Jul 14;9:927206. doi: 10.3389/fnut.2022.927206 (PMC9329686; doi:10.3389/fnut.2022.927206)
Supplement: Supplementary Table 1 — Composition and nutrient levels of experimental diets. [file Table_1.DOCX]

| Items | Groups | | |
| --- | --- | --- | --- |
|  | C50 | C65 | C80 |
| Ingredient |  |  |  |
| Oats hay | 50 | 35 | 20 |
| Corn | 25.77 | 28.65 | 30.09 |
| Wheat | 5.25 | 8.39 | 12.68 |
| Wheat bran | 5.05 | 8.76 | 12.94 |
| Rapeseed meal | 5.01 | 8.95 | 12.99 |
| Soybean meal | 2.67 | 3.51 | 4.21 |
| Palm oil powder^1^ | 2.25 | 2.74 | 3.09 |
| CaHPO_4_ | 1.00 | 1.00 | 1.00 |
| NaCl | 1.00 | 1.00 | 1.00 |
| Premix^2^ | 2.00 | 2.00 | 2.00 |
| Composition^3^ |  |  |  |
| ME/(Mcal/kg) | 2.52 | 2.64 | 2.76 |
| NEg/(Mcal/kg) | 1.02 | 1.12 | 1.21 |
| CP | 12.41 | 13.18 | 13.87 |
| NDF | 37.67 | 31.36 | 25.48 |
| ADF | 23.32 | 18.36 | 13.61 |
| Ca | 0.45 | 0.48 | 0.50 |
| P | 0.48 | 0.56 | 0.63 |

**Supplementary Table 1 Composition and nutrient levels of experimental diets (DM basis)**

^1^ ME of palm oil powder was 6.55 Mcal/kg, NEg of palm oil powder was3.62 Mcal/kg and the value referenced NRC (2016).

^2^ The premix provided the following per kg of diets; Cu 10mg, Fe 65mg, Mn 30mg, Zn 25mg, I 0.5mg, Se 0.1mg, Co 0.1mg, Vitamin A 4000IU, Vitamin D 500IU, Vitamin E 40IU.

^3^ME and NEg was a calculated value, while the others were measured values.
